# Supplementary figures and images for: MicNet toolbox: Visualizing and unraveling a microbial network
Source: PLoS One. 2022 Jun 24;17(6):e0259756. doi: 10.1371/journal.pone.0259756 (PMC9231805; doi:10.1371/journal.pone.0259756)

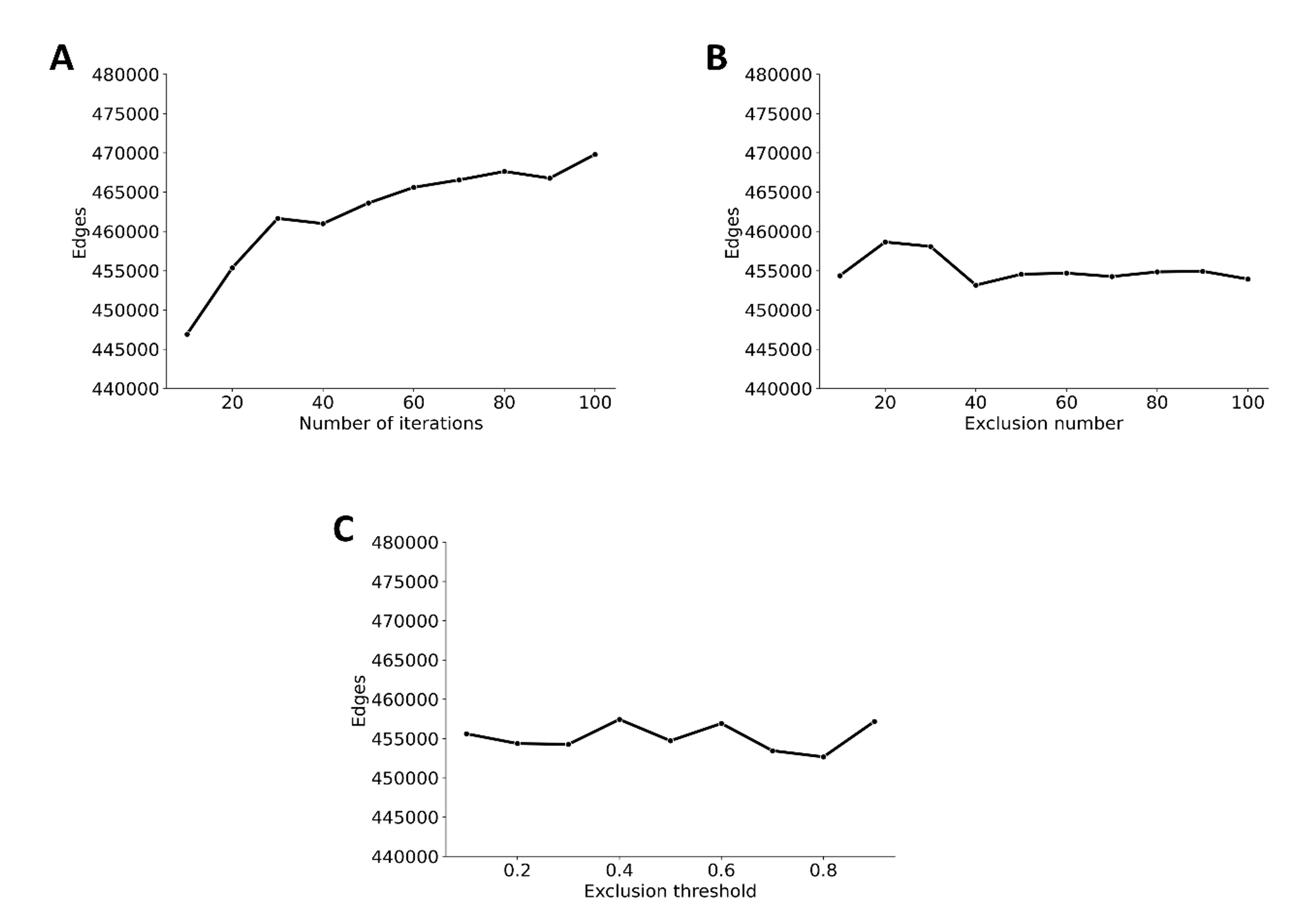

Supplement: S1 Fig — SparCC parameters were set based on our most complex network: Archean Domes. We ran SparCC varying A. the number of iterations from 10 to 100, B. the exclusion number from 10 to 100 and C. the exclusion threshold from 0.1 to 0.9. We chose the final values based on the stabilization of the number of edges found, such that the final values used for our databases were: 50 iterations, 10 exclusion number and 0.1 for exclusion threshold. (PNG) [file pone.0259756.s001.png]

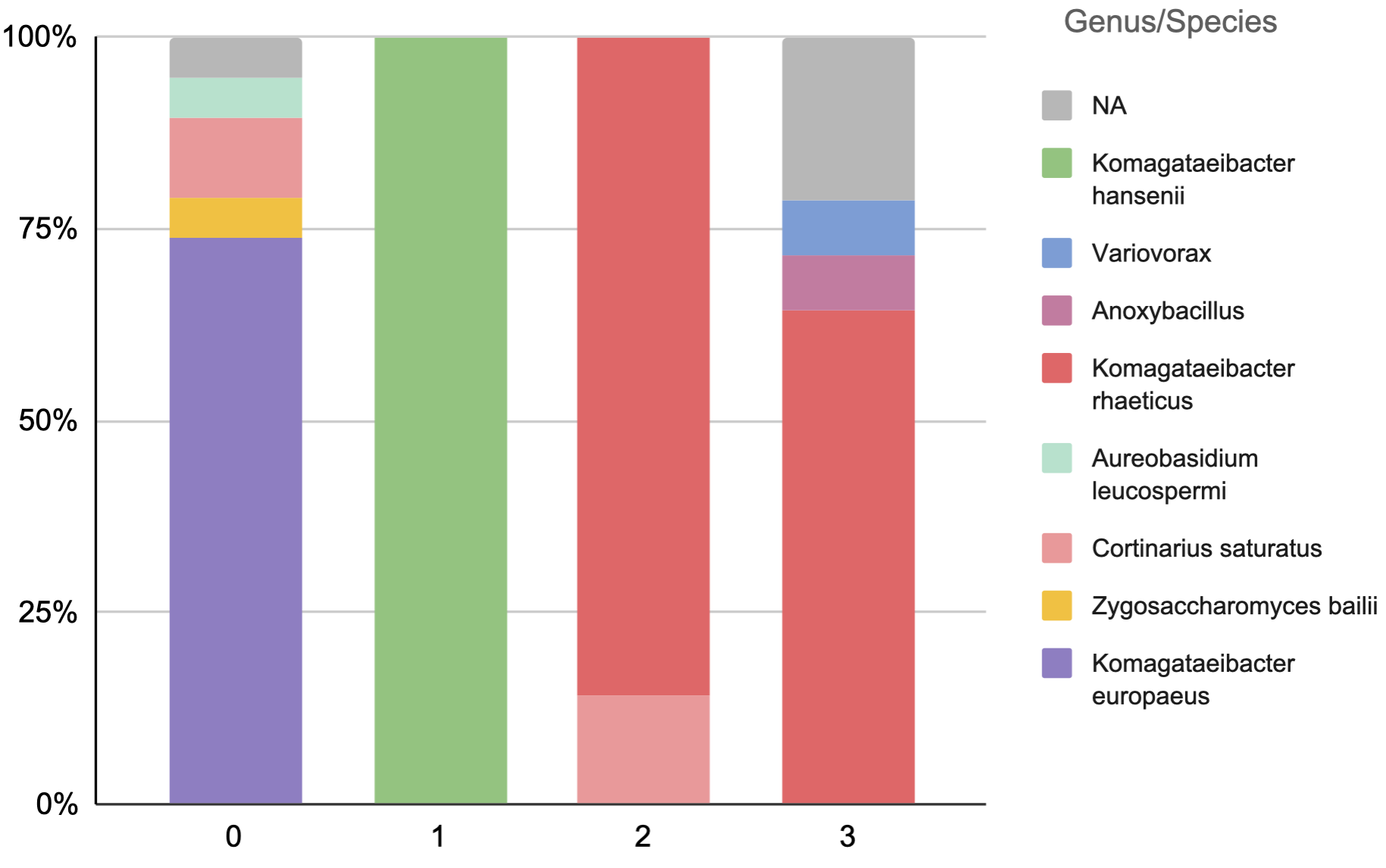

Supplement: S2 Fig — Community ID is shown in the x-axis. (PNG) [file pone.0259756.s002.png]

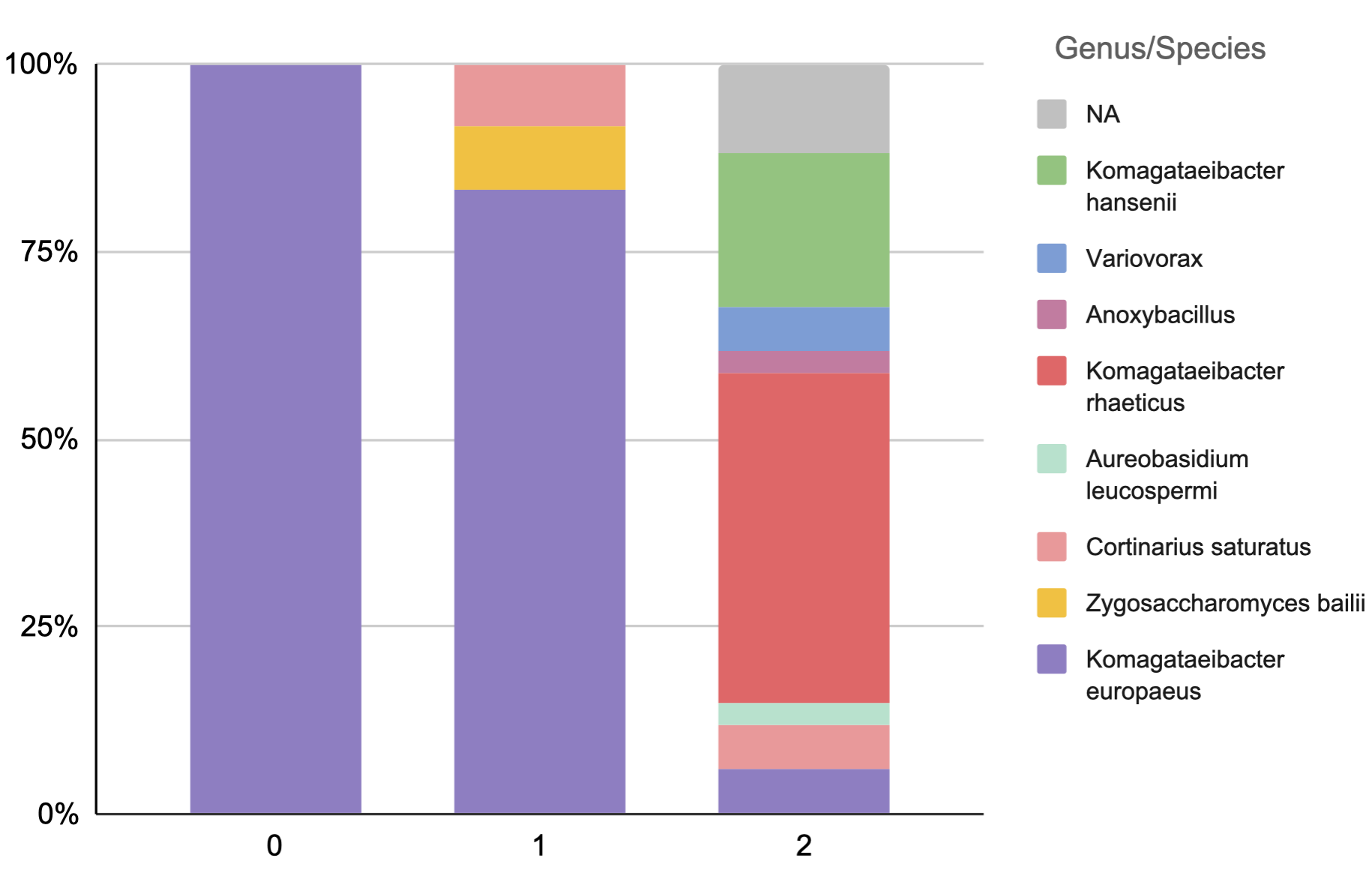

Supplement: S3 Fig — Cluster ID is shown in the x-axis. (PNG) [file pone.0259756.s003.png]

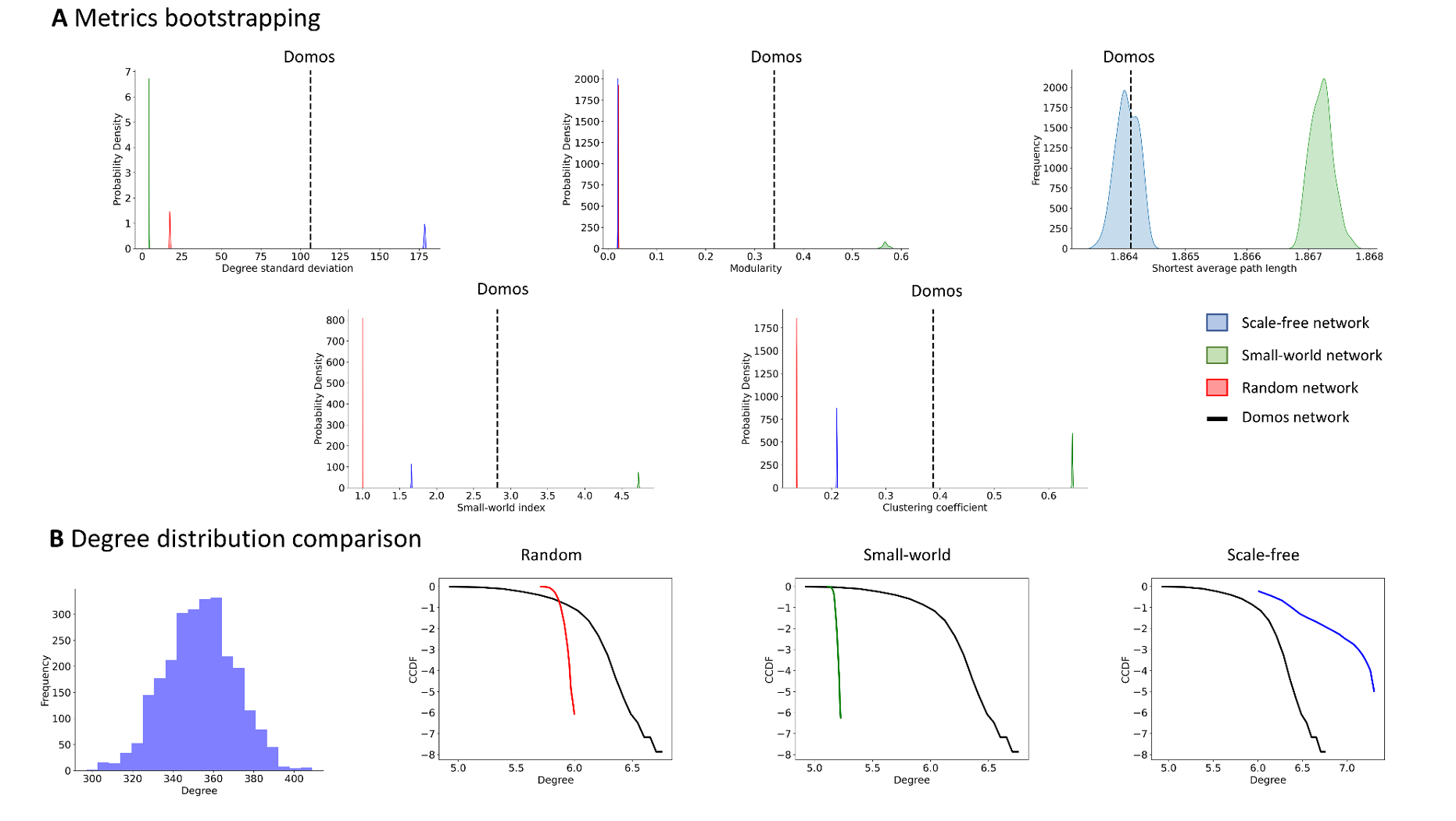

Supplement: S4 Fig — A. Distributions obtained from simulated random (red), scale-free (blue) and small-world (green) networks and its comparison to the metrics found in the Archean Domes network for: degree variance, modularity, average path length, small-world index, and clustering coefficient. B. Degree distribution of the Archean Domes network. We also show the comparison of the kombucha CCDF with a random network CCDF, a small-world network, and a scale-free network. (PNG) [file pone.0259756.s004.png]

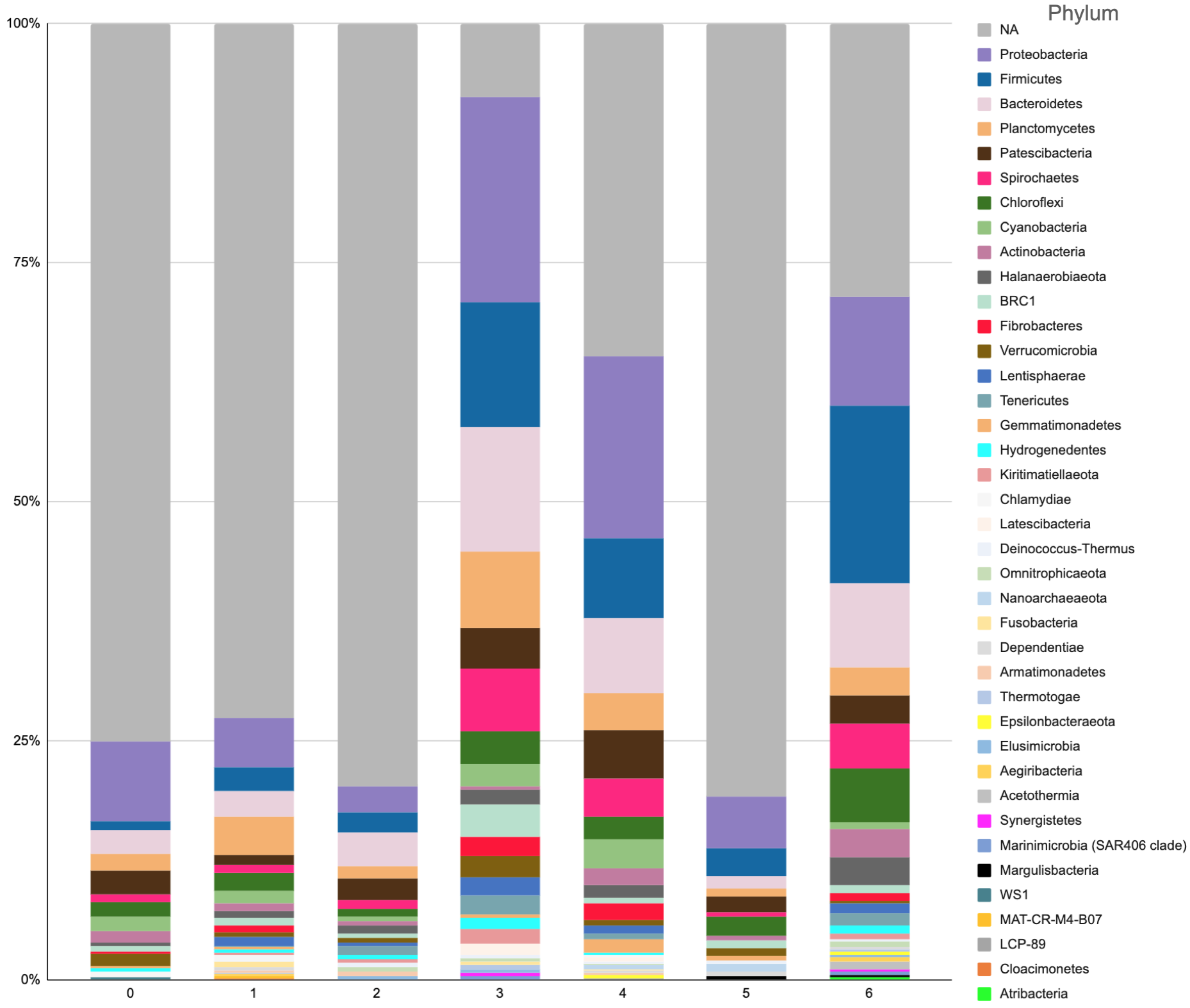

Supplement: S5 Fig — Community ID is shown in the x-axis. Unassigned bacteria and not annotated sequences are grouped in the NA category. (PNG) [file pone.0259756.s005.png]

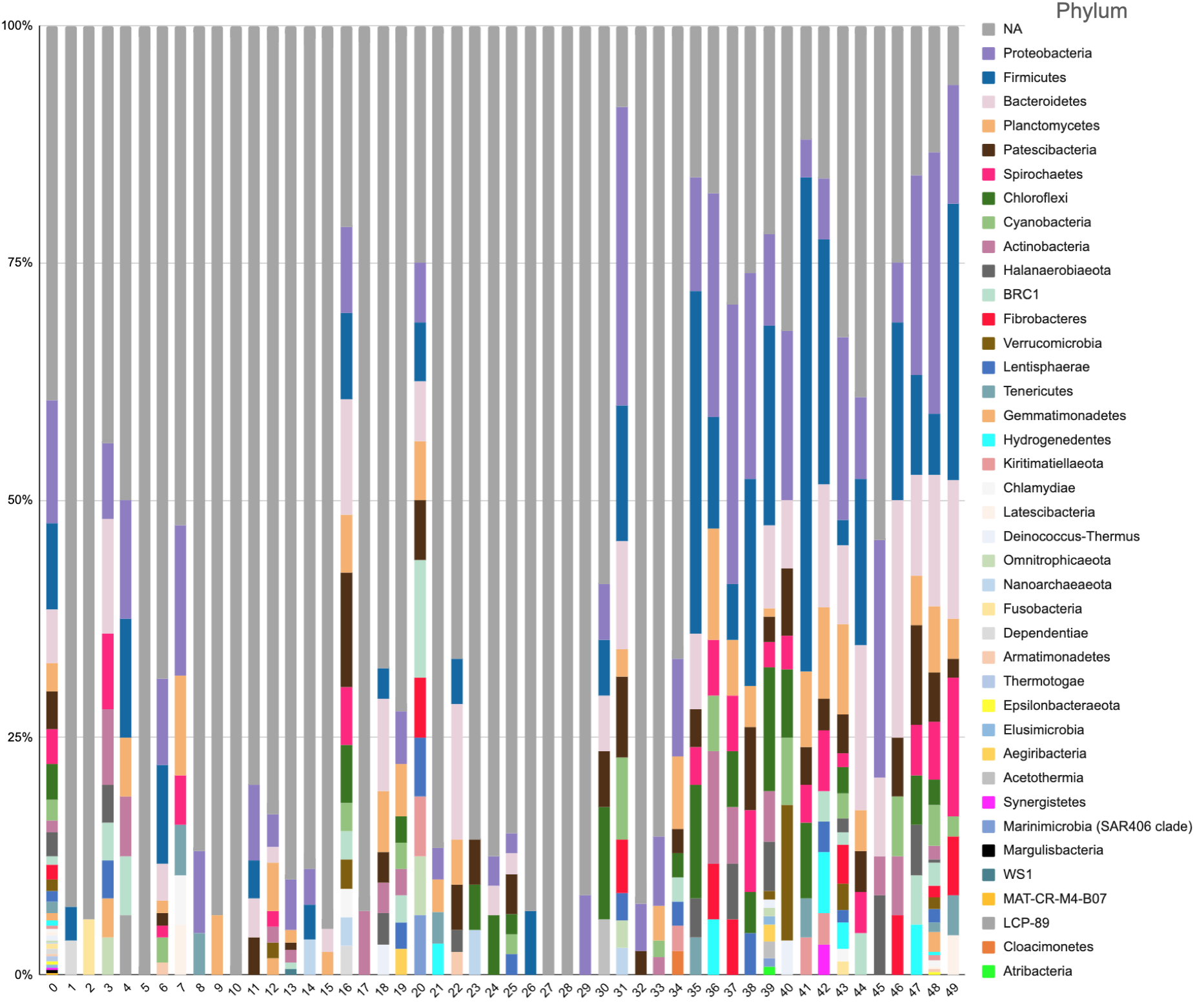

Supplement: S6 Fig — Cluster ID is shown in the x-axis. Unassigned bacteria and not annotated sequences are grouped in the NA category. (PNG) [file pone.0259756.s006.png]

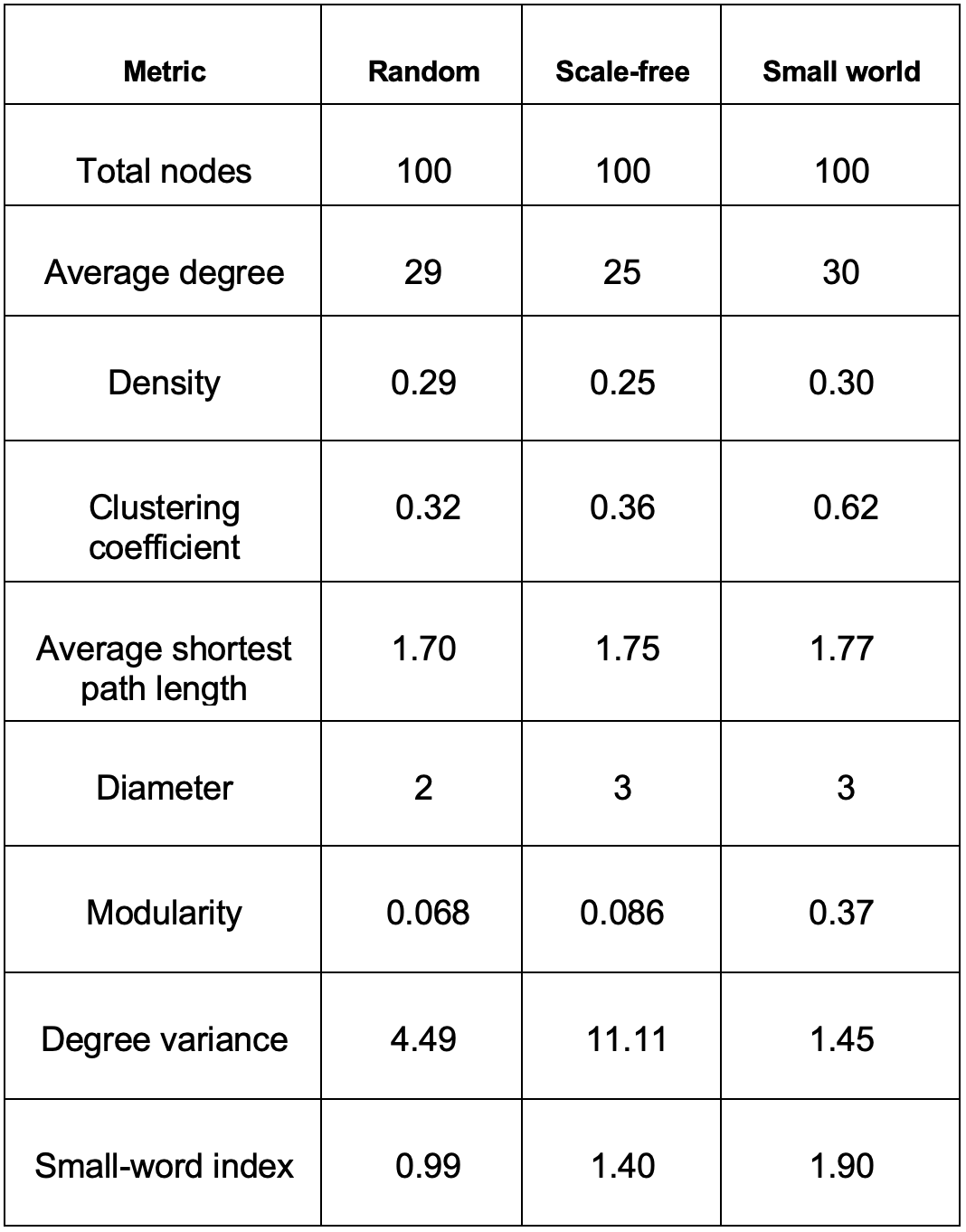

Supplement: S1 Table — Large scale metrics of three simulated networks with a random, scale-free and small world topology. (PNG) [file pone.0259756.s007.png]

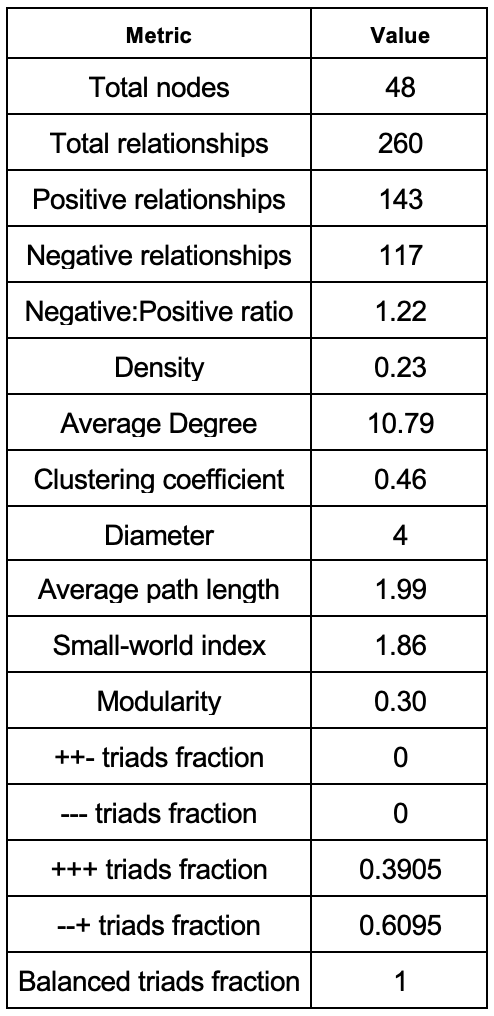

Supplement: S2 Table — (PNG) [file pone.0259756.s008.png]

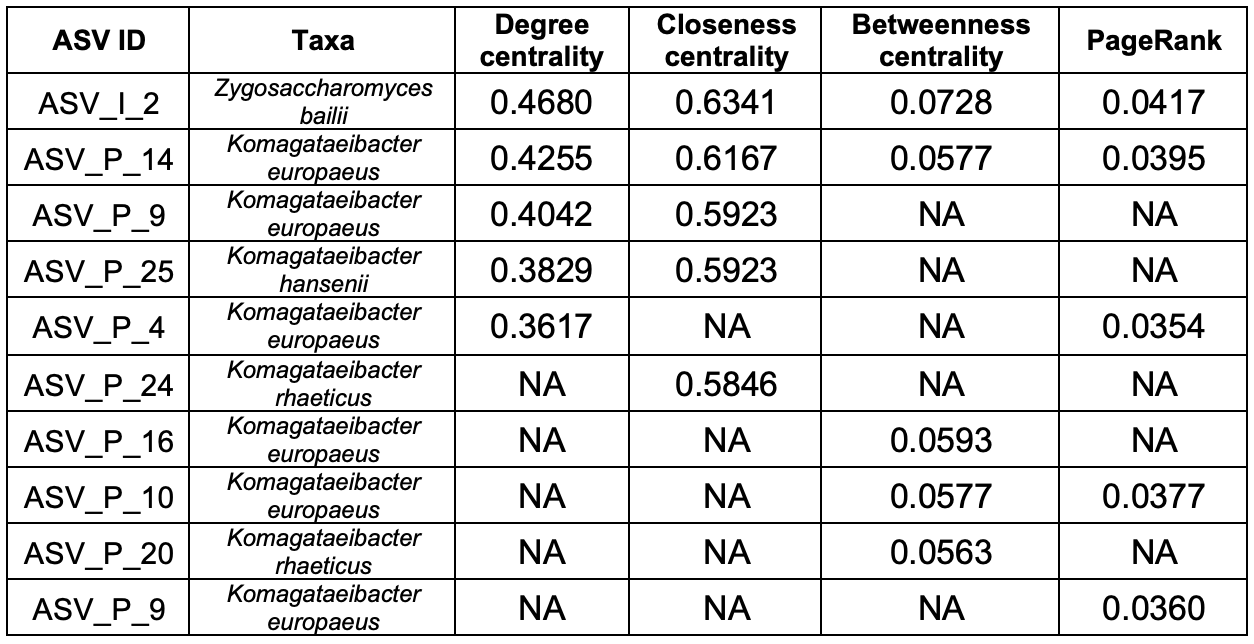

Supplement: S3 Table — Degree, closeness, betweenness, and PageRank centrality was calculated for the top 5 ASV respectively. If a given ASV was not among the top 5 in a centrality measure, the value is reported as NA. (PNG) [file pone.0259756.s009.png]

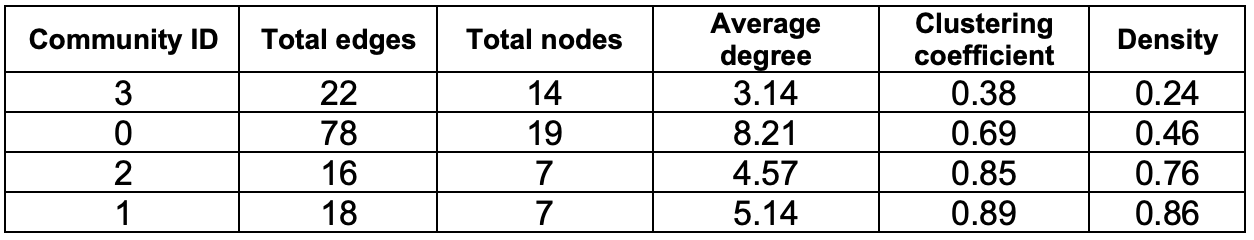

Supplement: S4 Table — For each community, total nodes, diameter, clustering coefficient, and average shortest path were calculated. Clusters are ordered by increasing density. (PNG) [file pone.0259756.s010.png]

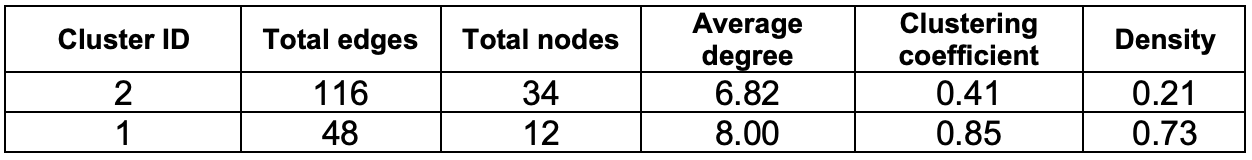

Supplement: S5 Table — For each community, total nodes, diameter, clustering coefficient, and average shortest path were calculated. Clusters are ordered by increasing density. (PNG) [file pone.0259756.s011.png]

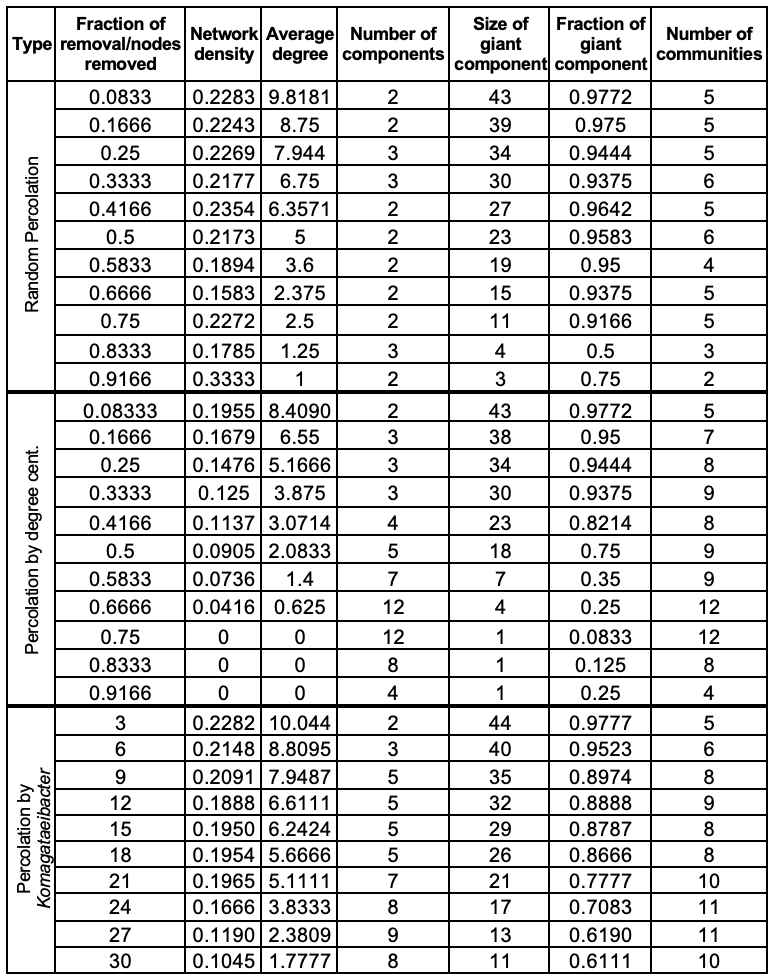

Supplement: S6 Table — Random, by groups (Genus), and by degree centrality percolation simulations were performed on the Louvain groups. For the percolation by groups, only Komagataeibacter and is shown. (PNG) [file pone.0259756.s012.png]

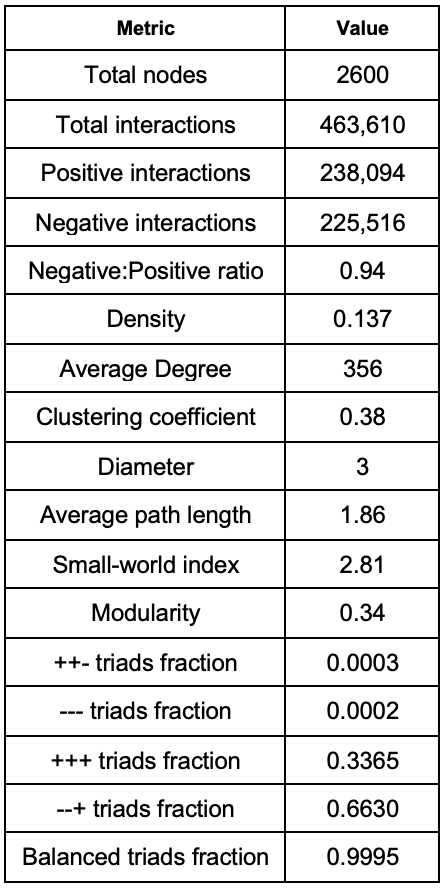

Supplement: S7 Table — (PNG) [file pone.0259756.s013.png]

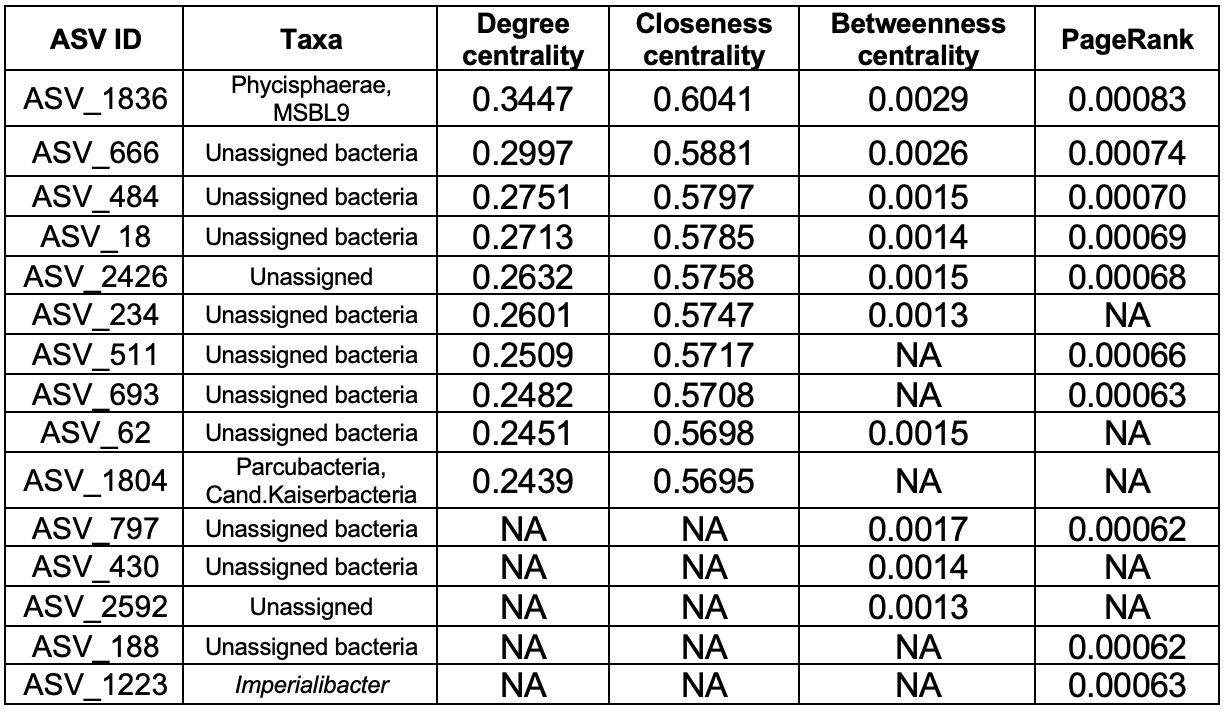

Supplement: S8 Table — Degree, closeness, betweenness, and PageRank centrality was calculated for the top 10 ASV respectively. If a given ASV was not among the top 10 in a centrality measure, the value is reported as NA. (PNG) [file pone.0259756.s014.png]

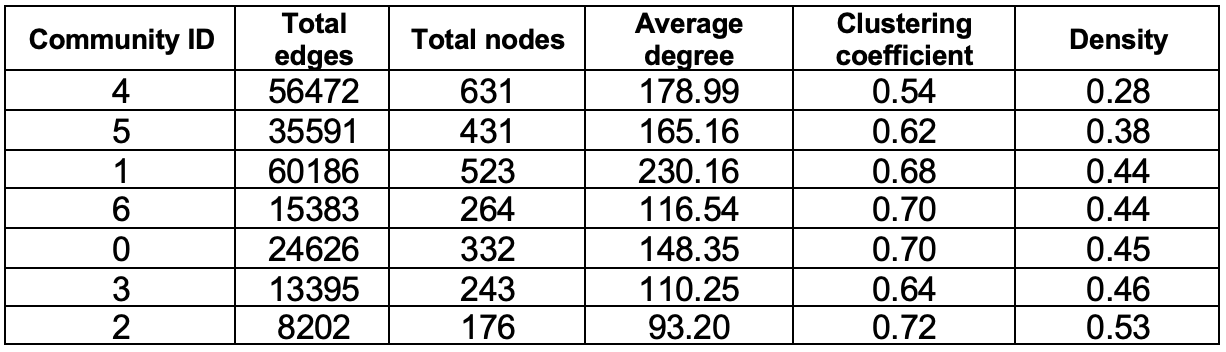

Supplement: S9 Table — For each community, total edges, total nodes, average degree, clustering coefficient, and density were calculated. Clusters are ordered by increasing density. (PNG) [file pone.0259756.s015.png]

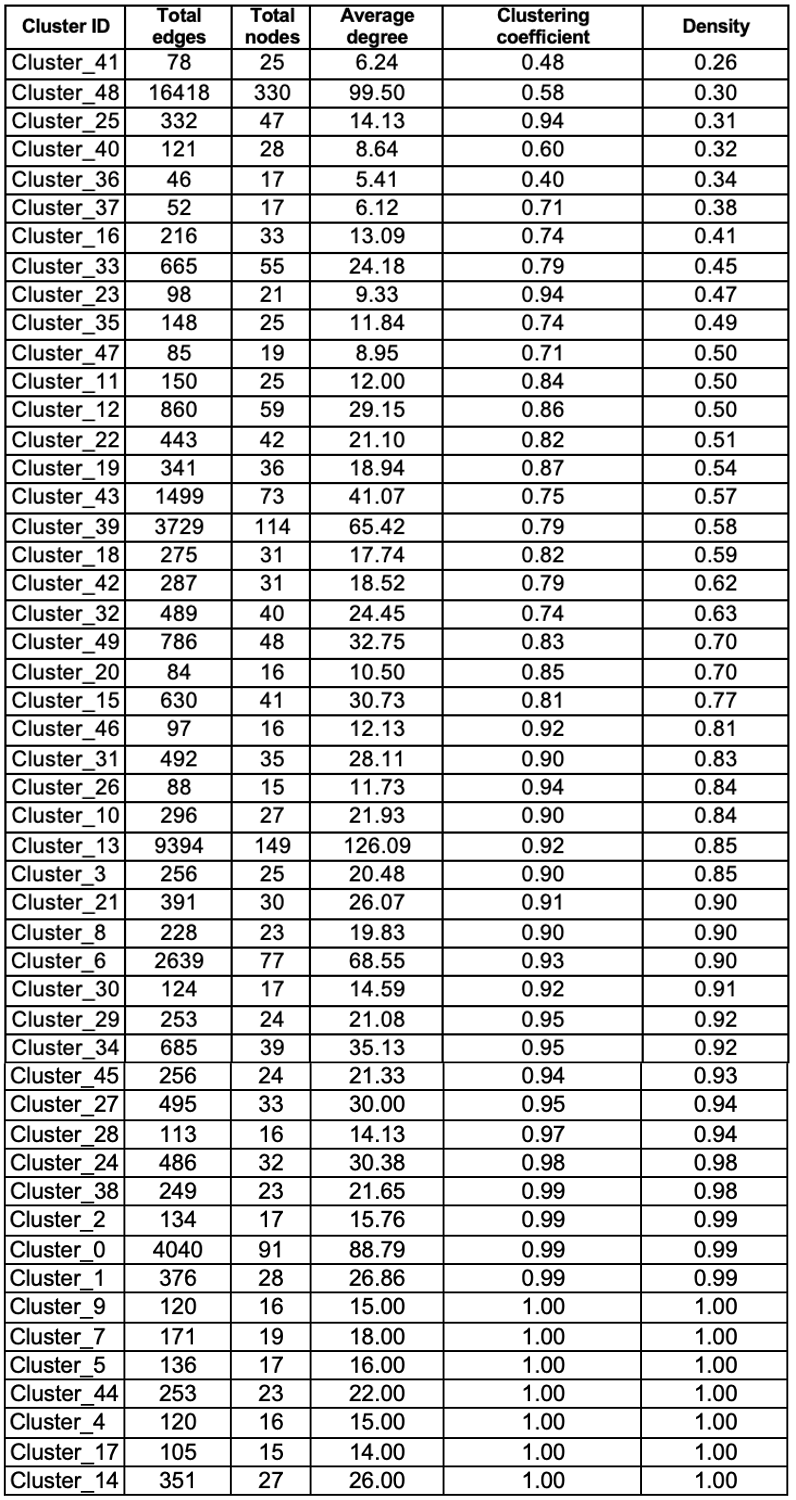

Supplement: S10 Table — For each community, total edges, total nodes, average degree, clustering coefficient, and density were calculated. Clusters are ordered by increasing density. (PNG) [file pone.0259756.s016.png]

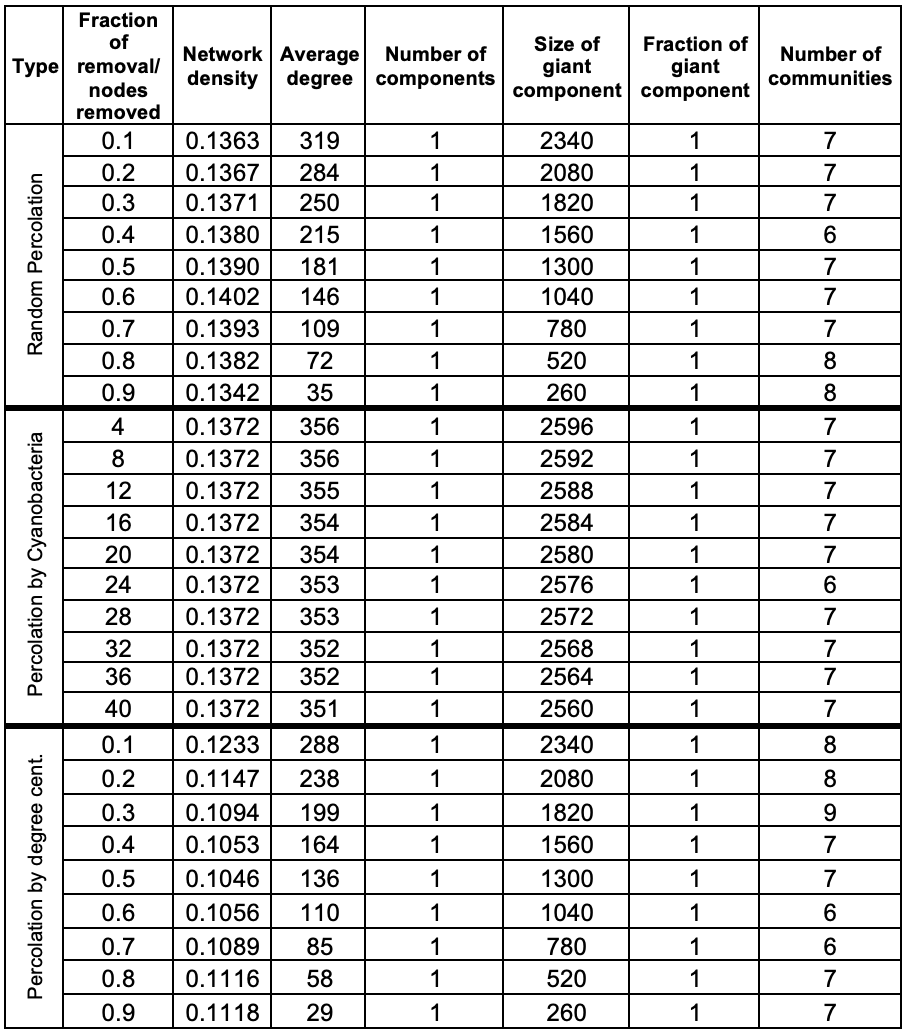

Supplement: S11 Table — Random, by groups (Phylum), and by degree centrality percolation simulations were performed on the Louvain groups. For the percolation by groups, only Cyanobacteria percolation is shown. (PNG) [file pone.0259756.s017.png]
